# Supplementary material for: Direct Photochemical Synthesis of Substituted Benzo[b]fluorenes
Source: Org Lett. 2024 Nov 25;26(48):10364–8. doi: 10.1021/acs.orglett.4c03978 (PMC11629382; doi:10.1021/acs.orglett.4c03978)
Supplement: Supplementary file 2 — ol4c03978_si_002.zip [file ol4c03978_si_002.zip › FID for Publication/5d/20240424_RC-2-C-5-69_01/plots/RC-2-C-5-69_CARBON_20240425_01_plot01.pdf]

RC-2-C-5-69

Location	3	Pulse sequence	CARBON	Temperature	25	Suboperator	RuairiCrawford
Date collected	2024-04-24	Solvent	dmso	Spectrometer	Agilent-NMR-vnmrs500	Operator	MBaumann

SAMPLE

date	Apr 24 2024
solvent	dmso
file	/home/data/MBaumann/newucd500/RuairiCrawford/2024/20240424_RC-2-C-5-69_01/RC-2-C-5-69_CARBON_20240425_01.fid

ACQUISITION

sw	31250.0
at	1.049
np	65536
fb	17000
bs	8
ss	40
d1	1.000
nt	5000
ct	5000

TRANSMITTER

tn	C13
sfrq	125.747
tof	1914.5
tpwr	58
pw	4.900

DECOUPLER

dn	H1
dof	0
dm	yyy
decwave	w
dpwr	41
dmf	12579

PRESATURATION

satmode	n
wet	n

SPECIAL

temp	25.0
gain	60
spin	0
hst	0.008
pw90	9.800
alfa	10.000

FLAGS

il	n
in	n
dp	y
hs	nn

PROCESSING

lb	0.50
fn	not used

DISPLAY

sp	-1794.4
wp	31250.0
rfl	1794.4
rfp	0
rp	24.7
lp	0

PLOT

wc	252
sc	8
vs	5611
th	50

ai cdc ph

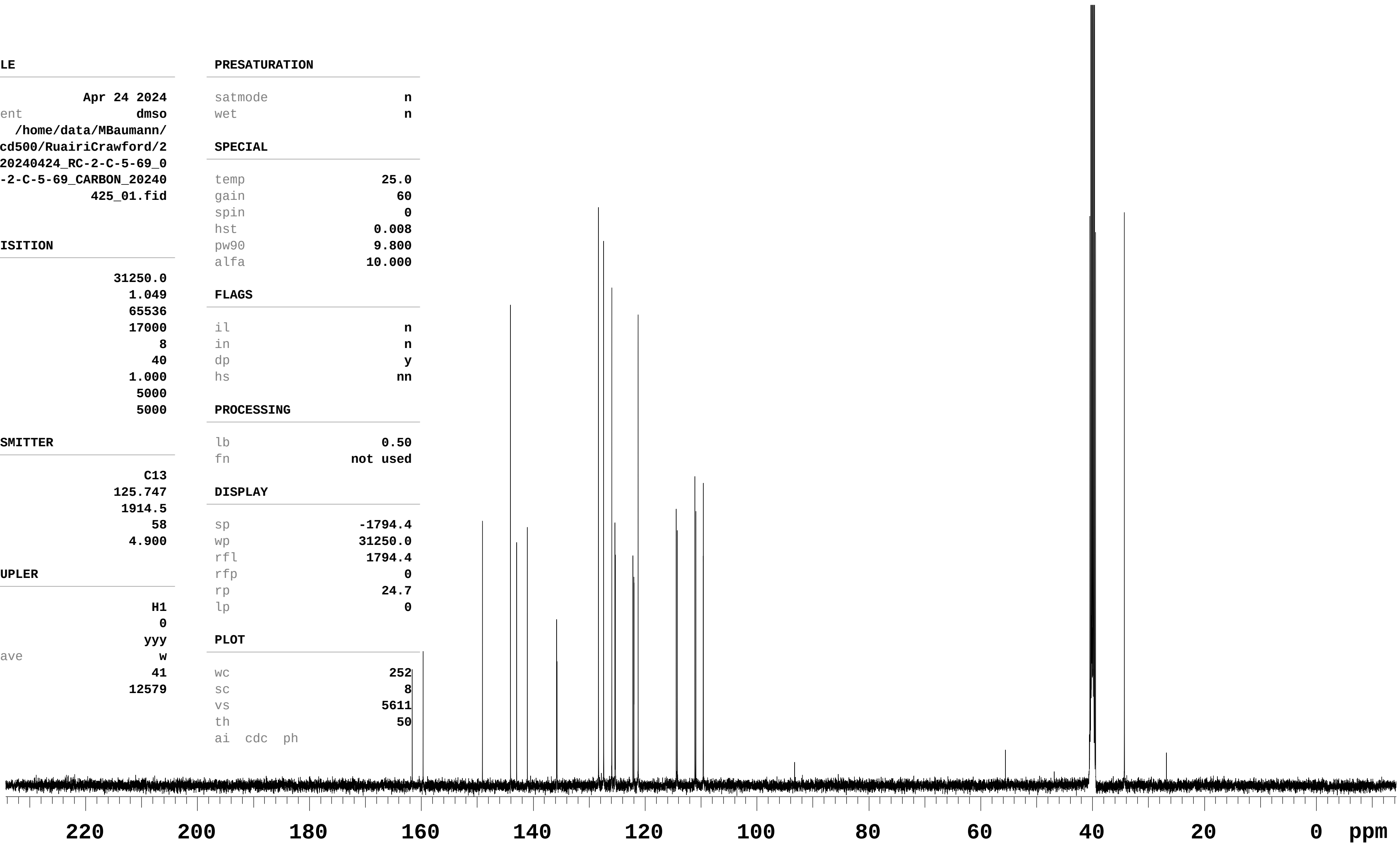

Plotname: RC-2-C-5-69_CARBON_20240425_01_plot01

Data file /home/data/MBaumann/newucd500/RuairiCrawford/2024/20240424_RC-2-C-5-69_01/RC-2-C-5-69_CARBON_20240425_01.fid

Plot date 2024-04-25
